# An emerging consensus for the structure of EmrE

**DOI:** 10.1107/S0907444908036640

**Published:** 2009-01-20

**Authors:** Vladimir M. Korkhov, Christopher G. Tate

**Affiliations:** a MRC Laboratory of Molecular Biology, Hills Road, Cambridge CB2 0QH, England

**Keywords:** EmrE, multidrug transporters, small multidrug-resistance family

## Abstract

The archetypical member of the small multidrug-resistance family is EmrE, a multidrug transporter that extrudes toxic polyaromatic cations from the cell coupled to the inward movement of protons down a concentration gradient. The architecture of EmrE was first defined from the analysis of two-dimensional crystals by cryoelectron microscopy (cryo-EM), which showed that EmrE was an unusual asymmetric dimer formed from a bundle of eight α-helices. The most favoured interpretation of the structure was that the monomers were oriented in opposite orientations in the membrane in an antiparallel orientation. A model was subsequently built based upon the cryo-EM data and evolutionary constraints and this model was consistent with mutagenic data indicating which amino-acid residues were important for substrate binding and transport. Two X-ray structures that differed significantly from the cryo-EM structure were subsequently retracted owing to a data-analysis error. However, the revised X-ray structure with substrate bound is extremely similar to the model built from the cryo-EM structure (r.m.s.d. of 1.4 Å), suggesting that the proposed antiparallel orientation of the monomers is indeed correct; this represents a new structural paradigm in membrane-protein structures. The vast majority of mutagenic and biochemical data corroborate this structure, although cross-linking studies and recent EPR data apparently support a model of EmrE that contains parallel dimers.

## Abstract 2

The ongoing story of the structure of EmrE, the archetypical member of the small multidrug-resistance family, is described.

## 1. Introduction

EmrE from *Escherichia coli* is the archetypical small multidrug-resistance (SMR) transporter and has been extensively studied using a multitude of techniques (Schuldiner *et al.*, 2001 ▶) since its initial characterization and the demonstration of its role as a multidrug transporter (Yerushalmi *et al.*, 1995 ▶). However, over the last five years it has engendered considerable debate for two main reasons. Firstly, two X-ray structures of EmrE (Ma & Chang, 2004 ▶; Pornillos *et al.*, 2005 ▶) were com­pletely different (Tate, 2006 ▶) from the previously determined cryo-EM structure (Ubarretxena-Belandia *et al.*, 2003 ▶); both X-ray structures were subsequently retracted owing to a data-handling error (Chang *et al.*, 2006 ▶). Secondly, the proposed arrangement of the monomers in the EmrE dimer in an antiparallel orientation, as suggested by both the cryo-EM (Ubarretxena-Belandia *et al.*, 2003 ▶) and X-ray structures (Ma & Chang, 2004 ▶; Pornillos *et al.*, 2005 ▶), was contradicted by biochemical and cross-linking studies (reviewed in Schuldiner, 2007*a* ▶,*b* ▶). Together, these discrepancies have clouded the field and, with the publication of a third X-ray structure (Chen *et al.*, 2007 ▶), it is now appropriate to review the biophysical and structural data and then discuss possible areas of conflict.

## 2. The oligomeric state of EmrE and the cryo-EM structure

When EmrE is purified using the detergent dodecylmaltoside (DDM), the resulting protein is able to bind the substrate tetraphenylphosphonium (TPP^+^) with a *K*_d_ of 2 n*M*, which is identical to the affinity of unpurified EmrE for TPP^+^ in *E. coli* membranes (Tate *et al.*, 2003 ▶). This suggests that the structure of the substrate-binding pocket is unperturbed during purification and is indicative that the overall structure is also unchanged. Sedimentation-equilibrium analytical ultracentri­fugation (AUC) unambiguously showed that this purified EmrE sample comprises a monomer and dimer in equilibrium, with no indication of higher oligomeric states (Butler *et al.*, 2004 ▶). These data correlate with analysis by size-exclusion chromatography (SEC) performed at 277 K, in which EmrE is predominantly a dimer that dissociates only very slowly to the monomer. Indeed, the dimer is remarkably stable in DDM and incubation at 353 K for 15 min is required to dissociate DDM-solubilized EmrE into monomers (Rotem *et al.*, 2001 ▶). The molar ratio of substrate binding to purified EmrE was determined to be one molecule of substrate per two molecules of EmrE by saturation-binding experiments using ^3^H-TPP^+^ and is consistent with EmrE being a dimer in detergent (Butler *et al.*, 2004 ▶; Tate *et al.*, 2003 ▶). Monomeric preparations of EmrE, as determined by AUC, have also been produced by purifying the protein in chloroform–methanol and resuspending the dried-down protein in DDM (Winstone *et al.*, 2005 ▶). It is remarkable that many planar substrates such as ethidium bind to this preparation with similar affinities to dimeric EmrE, presumably owing to the binding of the hydrophobic cations to Glu14, which is in a hydrophobic environment; in contrast, TPP^+^ bound with an affinity four orders of magnitude weaker than for EmrE purified in DDM (Sikora & Turner, 2005 ▶), indicating that monomeric EmrE is not in its native conformation. That EmrE is a dimer in detergent solution and represents the minimal functional unit for substrate binding is therefore undisputed. However, it is still unclear whether EmrE is a dimer **in vivo** or whether it forms a higher, perhaps tetrameric, oligomeric state (Ubarretxena-Belandia & Tate, 2004 ▶); the only data addressing this issue are from a negative dominance study suggesting that EmrE may form an oligomer larger than a dimer (Yerushalmi *et al.*, 1996 ▶).

**Figure 1:**
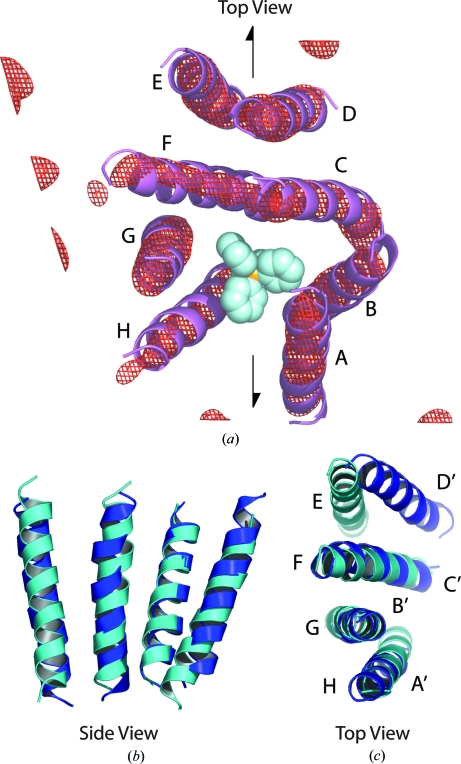
Structure of EmrE determined by cryo-EM at 7.5 Å resolution. (*a*) A view perpendicular to the membrane plane, with density contoured at 2σ (red mesh) to which α-helices were fitted by eye. TPP^+^ is represented by a space-filling model. Half arrows represent the in-plane pseudo-twofold axis that relates the two monomers. The superposition of helices *A*–*D* onto helices *H*–*E* after rotation by 160° about the twofold axis is shown from a side view (*b*) and top view (*c*). Reprinted from Tate (2006 ▶), with permission from Elsevier.

The first indications that the structure of EmrE was an unusual asymmetric dimer came from its projection structure determined by cryo-EM and image reconstruction of two-dimensional crystals (Tate *et al.*, 2001 ▶). TPP^+^ bound to these two-dimensional crystals with the same affinity as to detergent-solubilized EmrE and to unpurified EmrE in *E. coli* membranes, so it is likely that the two-dimensional crystals contained functional EmrE (Ubarretxena-Belandia & Tate, 2004 ▶). In fact, it was possible to elucidate that it was the EmrE molecules within the crystalline lattice that bound the TPP^+^ because there was a conformational change in the transporter that caused disruption of the crystalline lattice and altered the planar space group from *c*222 to *p*2 (Tate *et al.*, 2003 ▶). Crystals grown in the presence of TPP^+^ also had a *p*2 lattice and a comparison of the crystals grown in the absence or presence of TPP^+^ identified the site of TPP^+^ binding as a region surrounded by six of the eight helices forming the EmrE dimer (Tate *et al.*, 2003 ▶). The eight helices in the cryo-EM structure were labelled *A*–*H* in an anticlockwise manner in the view shown in Fig. 1 ▶(*a*) because it was not possible to assign the amino-acid sequence to the corresponding density at 7.5 Å resolution; in this nomenclature, the binding pocket is formed from helices *A*–*B*–*C* from one monomer and *H*–*G*–*F* from the other. The TPP^+^-binding pocket is also the site of binding of three planar substrates, although EmrE binds these planar substrates with a slightly different conformation from that of the TPP^+^ complex (Korkhov & Tate, 2008 ▶). Determination of the three-dimensional structure of EmrE from the two-dimensional crystals by cryo-EM confirmed the presence of density corresponding to TPP^+^ in the centre of a binding pocket bounded by six α-helices (Fig. 1 ▶; Ubarretxena-Belandia *et al.*, 2003 ▶).

**Figure 2:**
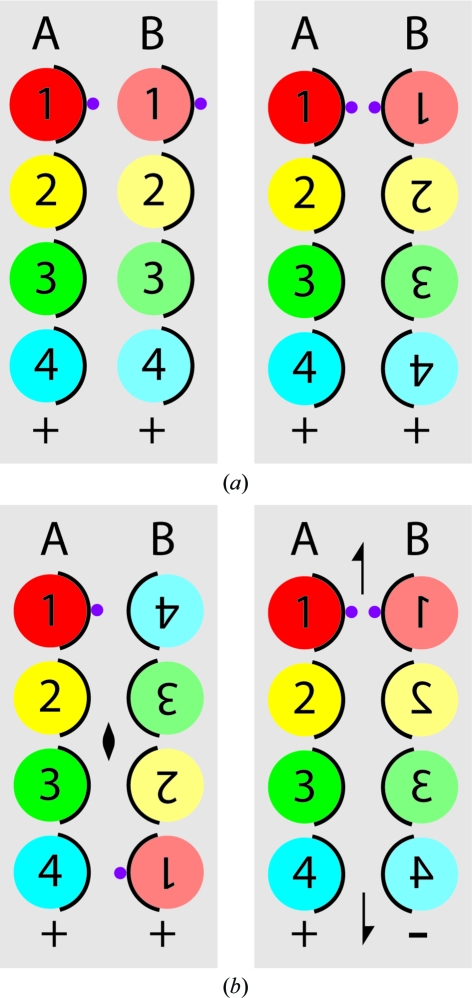
Theoretical considerations of how two identical monomers could be arranged to form a dimer. In each of the panels the EmrE dimer is viewed perpendicular to the membrane plane with each helix labelled 1–4 in a different colour. The conserved faces of each helix are depicted as an arc of black and the position of Glu14 is shown as a small purple sphere on helix 1; biochemical data indicate that both Glu14 residues must be closely juxtaposed. The relative topology of each monomer in the membrane is depicted by either a plus (+) or a minus (−) sign. The relationship between monomer *A* and monomer *B* is considered in terms of the transition required to go from *A* to *B*. (*a*) Parallel dimers related by a translation; this is unlikely given that conserved residues in *B* are oriented towards the lipid bilayer. (*b*) Parallel dimers related by translation followed by 180° rotation of each helix about its axis perpendicular to the membrane plane; this is unlikely given that the interfaces between the helices in monomer *A* are different from the helices in monomer *B*. (*c*) Parallel dimers related by a twofold axis perpendicular to the membrane plane; this is unlikely given that the two Glu14 residues are on opposite sides of the molecule. (*d*) Antiparallel dimers related by an in-plane twofold axis (half arrows); this is likely provided that the cell can synthesize a membrane protein with both orientations in the membrane, *i.e.* dual topology.

The cryo-EM structure of EmrE (Ubarretxena-Belandia *et al.*, 2003 ▶) has been called the ‘gold standard’ to which subsequent structures need to be compared (Rapp *et al.*, 2007*b* ▶) and it is only the interpretation of the structure that has been brought into question (Schuldiner, 2007*b* ▶). Although the assignment of the α-helices to specific amino-acid sequences was not possible, the striking presence of an in-plane pseudo-twofold axis relating helices *A*–*B*–*C* to *H*–*G*–*F* by a 160° rotation (Fig. 1 ▶) suggested the novel architecture consisting of antiparallel dimers (Ubarretxena-Belandia *et al.*, 2003 ▶; Tate, 2006 ▶). Indeed, even a brief consideration of possible models for how two identical monomers can pack together, based on our understanding of how transmembrane helices pack in other membrane proteins, leads inexorably to the conclusion that the most plausible model is composed of antiparallel dimers (Fig. 2 ▶). This is because conserved residues, which in membrane proteins point into the centre of the molecule to make specific helix–helix and helix–substrate interactions, will inevitably occur pointing towards the centre of the EmrE dimer (Fig. 2 ▶). Biochemical evidence supports this, because the highly conserved Glu14 residues from each monomer, which are both essential for transport, have to be in the binding pocket in close juxtaposition to perform substrate transport (Koteiche *et al.*, 2003 ▶; Muth & Schuldiner, 2000 ▶; Rotem *et al.*, 2001 ▶; Weinglass *et al.*, 2005 ▶; Yerushalmi *et al.*, 2001 ▶; Yerushalmi & Schuldiner, 2000 ▶). A model containing parallel monomers could in theory be possible, but completely new concepts in protein structure have to be invoked to explain it. The monomers in a parallel monomer model would have to be related by a translation, followed by a rotation of individual α-helices through 180° about the helical axis to place conserved residues in the centre of the dimer (Fig. 2 ▶). The consequence of this is that the interfaces between adjacent helices within each monomer would be completely different and would require the co-evolution of identical amino-acid sequences to make two different packing interfaces with similar efficiencies; this has never previously been found in any protein structure. In contrast, our understanding of the determinants of membrane-protein topology (von Heijne, 2006 ▶) offer ample precedent for a single membrane protein inserting into the membrane in two opposing orientations, both *in vivo* (Dunlop *et al.*, 1995 ▶) and also from model proteins with engineered topologies (Gafvelin & von Heijne, 1994 ▶). The fact that we do not understand fully the molecular details of how a single membrane protein can be inserted into the membrane in two different orientations does not in any way detract from the fact of their existence.

## 3. The X-ray structures of EmrE

**Figure 3:**
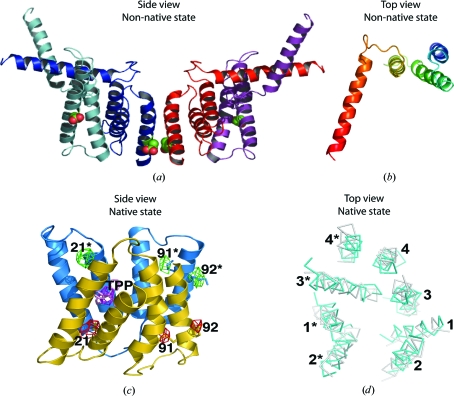
X-ray structures of EmrE. (*a*) The non-native structure of EmrE determined from crystals grown at pH 4.5 viewed parallel to the membrane plane with the positions of Glu14 shown as space-filling models and (*b*) the structure of one of the monomers viewed perpendicular to the membrane plane in rainbow coloration (N-terminus blue, C-terminus red); the structures are from PDB entry 1s7b, which has the same overall structure as the revised 3b61. (*c*) The corrected X-ray structure of EmrE containing bound TPP^+^ viewed parallel to the membrane plane (PDB code 3b5d). Electron density corresponding to Se from MAD data is shown as either a red mesh or green mesh depending upon the monomer in which the SeMet residues reside (numbered). The density corresponding to As in the tetraphenylarsonium substrate is shown as a purple mesh. (*d*) Comparison between the model based upon the cryo-EM structure and evolutionary constraints and the corrected X-ray structure. (*c*) and (*d*) are reprinted with permission from Chen *et al.* (2007 ▶), (Copyright 2007, National Academy of Sciences, USA).

The first two X-ray structures determined for EmrE (Ma & Chang, 2004 ▶; Pornillos *et al.*, 2005 ▶) did not correspond to the cryo-EM structure and were both proposed to be non-native (Tate, 2006 ▶); both X-ray structures had an incorrectly assumed hand and were subsequently retracted (Chang *et al.*, 2006 ▶). Recently, two revised structures have been published (Chen *et al.*, 2007 ▶). The structure of EmrE at pH 4.5 in the absence of substrate (PDB code 3b61) is very similar to the original structure and still represents a non-native state (Fig. 3 ▶). However, the recalculated X-ray structure of EmrE with TPP^+^ bound (PDB code 3b5d), including data from new crystals, fits extremely well into the density for the cryo-EM structure (Chen *et al.*, 2007 ▶). At 3.8 Å resolution it was not possible to build unambiguous models for the side chains, so only the C^α^ coordinates have been deposited. Confidence in the veracity of the structure comes from clear densities for Se from the MAD data sets used to obtain phases; it is particularly striking that pairs of densities for the SeMet residues in the dimer are entirely consistent with an antiparallel orientation of the monomers (Fig. 3 ▶). In addition, a model derived from the cryo-EM structure and evolutionary constraints (Fleishman *et al.*, 2006 ▶) has an r.m.s.d. of 1.4 Å compared with the C^α^ positions in the revised X-ray structure (Fig. 3 ▶). Finally, all the residues that have been predicted to be important in substrate binding and translocation are within the substrate-binding pocket delineated by the new X-ray structure (Chen *et al.*, 2007 ▶). Thus, there is now excellent agreement between the cryo-EM model and the 3.8 Å resolution structure derived from X-ray crystallo­graphy, showing that EmrE is an antiparallel dimer.

The non-native pH 4.5 structure (Chen *et al.*, 2007 ▶) is also interesting as it may represent the minimal energy fold of helices 1–3 immediately after synthesis *in vivo*, although in this scenario helix 4 would adopt a mobile trans­bilayer orientation rather than making contacts between neighbouring crystallo­graphic tetramers as it does in the crystal. The extremely close packing between the two helix 4s in the EmrE dimer suggests that it may provide the major driving force for dimerization and confer stability to the dimer during the conformational changes in the transport cycle.

## 4. Studies of EmrE homologues

**Figure 4:**
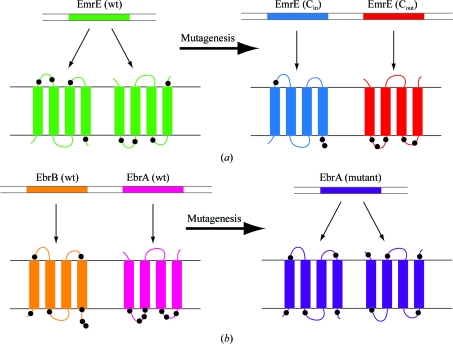
Orientation of SMR proteins in the membrane and experimental evidence for antiparallel dimers by mutagenesis. (*a*) EmrE is proposed to be an antiparallel homodimer in the membrane. Mutagenesis of positively charged residues (black circles) resulted in two genes expressing EmrE mutants with defined topology, either C_in_ or C_out_. Neither monomer was active on its own, but co-expression resulted in functionality (Rapp *et al.*, 2007*a* ▶). (*b*) EbrAB is a heterodimer that is only functional when both genes are expressed. Mutagenesis of EbrA to equalize the positively charged residues (black circles) on both faces of the membrane resulted in a functional homodimer (Kikukawa *et al.*, 2006 ▶).

Members of the SMR family homologous to EmrE are found widely throughout the bacterial world (Paulsen *et al.*, 1996 ▶). However, there are two different forms in which the homologues occur in bacteria. Firstly, a single gene can produce a functional homodimer, as is the case for *E. coli* EmrE (Yerushalmi *et al.*, 1995 ▶) and Smr from *Staphylococcus aureus* (Grinius & Goldberg, 1994 ▶). Secondly, a number of homologues are composed of heterodimers (Jack *et al.*, 2000 ▶; Masaoka *et al.*, 2000 ▶). The orientation that a bacterial membrane pro­tein adopts in the membrane can be predicted with reasonable accuracy by counting the number of Arg and Lys residues on one side of a membrane protein compared with the other; the face that has the greatest number of positively charged residues is on the cytoplasmic face of the membrane (the ‘positive inside’ rule; von Heijne, 1986 ▶). Comparisons of the charge distributions in the two different groups of EmrE homologues are extremely interesting (Fig. 4 ▶). In the case of *E. coli* EmrE, the distribution of Lys and Arg residues is fairly even between the two hydrophilic faces of the protein, which is in line with the prediction that it could be oriented in the membrane with dual topology, *i.e.* some of the molecules have intracellular N- and C-termini whilst others have extracellular N- and C-termini; this would be the case for the formation of antiparallel dimers. In contrast, homologues that are only functional as heterodimers are composed of monomers that have distinctive charge distributions, suggesting that each monomer can orient itself in the membrane only in one orientation, as is normal for the majority of membrane proteins. Each heterodimer is thus formed of one protein with N- and C-termini in the cytoplasm and one protein with N- and C-termini in the periplasm, *i.e.* they form antiparallel dimers.

Is there experimental evidence supporting the topological assignments predicted using the positive inside rule? The first clue to the abnormal topology of SMR proteins came from a global topology analysis of 700 inner membrane proteins from *E. coli* (Daley *et al.*, 2005 ▶). This was performed by fusing two topological reporters (GFP and PhoA) to the C-termini of all the proteins and assaying for either GFP or PhoA activity; if the C-terminus of the test protein normally resided in the cytoplasm this would lead to high GFP activity and low PhoA activity and *vice versa* for C-termini that resided in the periplasm. The results for SMR proteins did not fit this pattern and the suggestion was raised that they could all have dual topology (Rapp *et al.*, 2006 ▶). This was tested for EmrE by an elegant experiment that converted the normal EmrE homodimer into a heterodimer composed of two monomers of defined topological orientation (Rapp *et al.*, 2007*a* ▶); this was achieved by changing the number of positively charged residues on each face of the protein (Fig. 4 ▶). An *in vivo* assay for EmrE activity showed that each of the modified monomers of defined topology were inactive when expressed alone, but when they were expressed together normal EmrE activity was restored. This experiment shows that only antiparallel dimers are functional and that if EmrE monomers are all oriented in the membrane in the same fashion then EmrE cannot function. The corollary experiment (Fig. 4 ▶) has also been per­formed, in which a normally heterodimeric SMR family member, EbrAB, was evolved to function as a homodimer by removing the charge bias between the two faces of the protein (Kikukawa *et al.*, 2006 ▶). The topology of EbrAB was also tested using a Cys-labelling strategy with Cys residues engineered in the loops and at the N- and C-termini; all the constructs giving normal rates of substrate transport were found to adopt a topology predicted by the positive inside rule (Kikukawa *et al.*, 2007 ▶). In a concurrent series of experiments, a Cys-labelling strategy was employed to probe the topology of a series of EmrE mutants and the conclusion was that EmrE adopted an antiparallel orientation in the membrane (Nara *et al.*, 2007 ▶). All these experiments suggest that the SMR proteins tested to date function as antiparallel dimers.

## 5. Parallel *versus* antiparallel orientation of monomers in EmrE

The structural studies on *E. coli* EmrE and the topology studies mentioned above all seem to concur that the dimer is composed of monomers arranged in an antiparallel fashion, yet there are five papers to date that conclude the opposite, *i.e*. that EmrE is composed of monomers arranged in a parallel fashion with the N- and C-­termini pro­bably residing intracellularly (reviewed in Schuldiner, 2007*b* ▶). The techniques that have been used in these studies are varied and include cross-linking (Sos­kine *et al.*, 2002 ▶, 2006 ▶), EPR (McHaou­rab *et al.*, 2008 ▶), the construction of genetically fused EmrE holodimers (Steiner-Mordoch *et al.*, 2008 ▶) and topological studies using labelling strategies and the accessibility of tags to proteases (Ninio *et al.*, 2004 ▶). On the face of it, the use of multiple biochemical techniques leading to an apparently consistent conclusion is rather compelling, but this is opposed by equally compelling structural and biochemical data con­cluding the exact opposite. Can these views be reconciled or are there com­monalities in how experiments were performed that could give rise to erroneous conclusions?

Before trying to untangle this web of experiments, it must be appreciated that EmrE behaves abnormally compared with other membrane proteins. This was clear from the outset, where the first publication on the biochemistry of EmrE showed that it could be purified by extraction into chloroform–methanol solution and then reconstituted back into a functional form in proteoliposomes by drying it down in the presence of excess lipids (Yerushalmi *et al.*, 1995 ▶). This methodology was adapted to prepare monomeric EmrE in DDM (Winstone *et al.*, 2005 ▶) which was able to bind planar substrates with similar affinities to native EmrE, but crucially the high-affinity substrate TPP^+^ bound four orders of magnitude more weakly than to dimeric EmrE purified in DDM using standard techniques in aqueous buffers (Sikora & Turner, 2005 ▶). Thus, EmrE can exist in solution in a stable non-native conformation in a mild detergent; this is unusual in that most membrane proteins require harsh detergents such as SDS to maintain a similarly misfolded state. What is even more remarkable is that a non-native state of EmrE has actually been crystallized and its structure has been determined (Chen *et al.*, 2007 ▶). Normally, we assume that a misfolded protein will exist in multiple conformations that preclude crystallization, but apparently EmrE can exist predominantly in a single non-native conformation. As mentioned above, it is tempting to speculate that this non-native state could represent a state of the monomer in the membrane immediately after expression, with the final conformations of the monomer only being attained after dimerization.

The peculiarities of EmrE may also extend to the overproduction of the protein for structural and biochemical studies. If EmrE is indeed an antiparallel dimer, then its synthesis is probably a delicate balance between the production of the two orientations in the translocon which is based upon the balance of positive charges on the two soluble faces of the protein and the proton motive force present across the cellular membrane in *E. coli* (von Heijne, 2006 ▶; White & von Heijne, 2008 ▶). Anything that adversely affects either the folding pathway for the polypeptides or the overall energy balance of the cell could adversely affect the efficient production of EmrE. This is indeed what we have observed and has been noted by others in the production of native EmrE (Chen *et al.*, 2007 ▶). Extensive efforts were made to show that the purified EmrE used for the production of two-dimensional crystals was in a native conformation and that the EmrE in the two-dimensional lattice was also fully functional (Butler *et al.*, 2004 ▶; Tate *et al.*, 2001 ▶, 2003 ▶; Ubarretxena-Belandia & Tate, 2004 ▶); similar data have also now been published for the production of EmrE for three-dimensional crystallization (Chen *et al.*, 2007 ▶).

**Figure 5:**
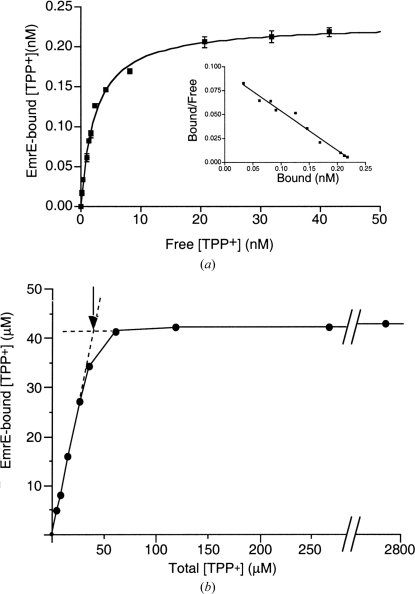
(*a*) Saturation binding curve of purified EmrE in detergent solution. *K*_d_ values were determined by nonlinear regression using a single-site model as indicated by the linearity of the Scatchard plot (inset). (*b*) Determination of the ratio of TPP^+^ binding to EmrE. The concentration of EmrE in the experiment was determined by amino-acid analysis to be 80.5 µ*M* and the intersection between the linear portions of the graph occurs at a TPP^+^ concentration of 40 µ*M*. The negligible increase in binding at 2.8 m*M* EmrE implies that there are no significant amounts of misfolded EmrE present in the purified sample. Reprinted from Tate *et al.* (2003 ▶), with permission from Elsevier.

What are the minimal data required to show that EmrE is indeed in a native state? Two things have to be shown. Firstly, that the affinity of binding of substrates is in the same range as for native EmrE in the membrane (Fig. 5 ▶). TPP^+^ is a good choice for this experiment because it is commercially available in a tritiated form, it binds to native EmrE with high affinity and there is a clear difference in binding between native (*K*_d_ ≃ 2 n*M*; Tate *et al.*, 2003 ▶) and non-native EmrE (*K*_d_ = 25 µ*M*; Sikora & Turner, 2005 ▶). Moreover, assays can be performed at high concentrations of TPP^+^ (Butler *et al.*, 2004 ▶) to exclude the possibility that the purified EmrE contains a small proportion of inactive protein. Binding assays performed at a single ligand concentration are insufficient because this will not give any indication of the affinity of binding. Secondly, the amount of potentially misfolded EmrE must be determined. For purified EmrE, this is trivial. The *B*_max_ from the saturation binding curve should equal half the number of moles of EmrE in solution as determined by amino-acid analysis (one ^3^H-­TPP^+^ binds per EmrE dimer). The molar ratio of ligand bound to EmrE can also be determined from a plot of ligand bound to EmrE *versus* the ligand concentration (Fig. 5 ▶). The advantage of this methodology is that high concentrations of EmrE can be used, which prevents any dissociation of the dimer, and this method is compatible with the use of either radioactive (Tate *et al.*, 2003 ▶) or fluorescent ligands (Chen *et al.*, 2007 ▶). If the EmrE sample is not purified, then the amount of misfolded EmrE can be assessed by using a dot-blotting technique (Zeder-Lutz *et al.*, 2006 ▶). Here, the total amount of tagged EmrE would be determined by comparison of the signal developed between a sample and a series of standards of known amounts of protein tagged with the identical epitope.

However it is determined, the amount of misfolded EmrE and the *K*_d_ for substrate binding of any EmrE sample has to be assessed before any biochemical experiment can be correctly evaluated. Unfortunately, this has never been determined in any experiment proposing the presence of parallel EmrE dimers. In some instances, *K*_d_ values for mutants have been determined but the effect of the point mutations on the ability of EmrE to fold efficiently has not been determined. As single point mutations can dramatically reduce the amount of protein expressed (Mordoch *et al.*, 1999 ▶), there can clearly be effects on the folding/stability of EmrE and the presence of misfolded protein is a real concern. Clearly, if most (*e.g.* 90%) of EmrE in a sample is incorrectly folded, then erroneous results are inevitable regardless of how carefully the rest of the experiments are performed. It is therefore entirely plausible that the experiments implying the existence of parallel monomers in the EmrE dimer are based on the topological characterization of misfolded protein and not native EmrE.
